# Increased mortality after a first myocardial infarction in human immunodeficiency virus-infected patients; a nested cohort study

**DOI:** 10.1186/s12981-015-0045-z

**Published:** 2015-02-22

**Authors:** David Carballo, Cécile Delhumeau, Sebastian Carballo, Caroline Bähler, Dragona Radovanovic, Bernard Hirschel, Olivier Clerc, Enos Bernasconi, Dominique Fasel, Patrick Schmid, Alexia Cusini, Jan Fehr, Paul Erne, Pierre-Fréderic Keller, Bruno Ledergerber, Alexandra Calmy, for the Swiss HIV Cohort Study and AMIS registry

**Affiliations:** Department of Cardiology, University Hospital, Geneva, Switzerland; HIV Metabolic Clinic, University Hospital, Geneva, Switzerland; General Internal Medicine, University Hospital, Geneva, Switzerland; AMIS Plus Data Center, Institute of Social and Preventive Medicine, University of Zurich, Zurich, Switzerland; Infectious Diseases, University Hospital Center, Lausanne, Switzerland; Infectious Diseases, Regional Hospital, Lugano, Switzerland; HIV Unit, Infectious diseases, University Hospital, Basel, Switzerland; Infectious Diseases, University Hospital, Sankt Gallen, Switzerland; Infectious Diseases, University Hospital, Bern, Switzerland; Division of Infectious Diseases and Hospital Epidemiology, University Hospital, Zürich, University of Zurich, Zürich, Switzerland; Cardiology Department, Peripherique Hospital, Lucern, Switzerland; Centre médical Ziggurat, Rue du Jura 11, 2900 Porrentruy, Switzerland

**Keywords:** Coronary artery disease, Myocardial infarction, HIV infection, Fatal outcome

## Abstract

**Aims:**

HIV infection may be associated with an increased recurrence rate of myocardial infarction. Our aim was to determine whether HIV infection is a risk factor for worse outcomes in patients with coronaray artery disease.

**Methods:**

We compared data aggregated from two ongoing cohorts: (i) the Acute Myocardial Infarction in Switzerland (AMIS) registry, which includes patients with acute myocardial infarction (AMI), and (ii) the Swiss HIV Cohort Study (SHCS), a prospective registry of HIV-positive (HIV+) patients. We included all patients who survived an incident AMI occurring on or after 1st January 2005. Our primary outcome measure was all-cause mortality at one year; secondary outcomes included AMI recurrence and cardiovascular-related hospitalisations. Comparisons used Cox and logistic regression analyses, respectively.

**Results:**

There were 133 HIV+, (SHCS) and 5,328 HIV-negative [HIV-] (AMIS) individuals with incident AMI. In the SHCS and AMIS registries, patients were predominantly male (72% and 85% male, respectively), with a median age of 51 years (interquartile range [IQR] 46–57) and 64 years (IQR 55–74), respectively. Nearly all (90%) of HIV+ individuals were on successful antiretroviral therapy. During the first year of follow-up, 5 (3.6%) HIV+ and 135 (2.5%) HIV- individuals died. At one year, HIV+ status after adjustment for age, sex, calendar year of AMI, smoking status, hypertension and diabetes was associated with a higher risk of death (HR 4.42, 95% CI 1.73-11.27). There were no significant differences in recurrent AMIs (4 [3.0%] HIV+ and 146 [3.0%] HIV- individuals, OR 1.16, 95% CI 0.41-3.27) or in hospitalization rates (OR 0.68 [95% CI 0.42-1.11]).

**Conclusions:**

HIV infection was associated with a significantly increased risk of all-cause mortality one year after incident AMI.

## Introduction

Coronary artery disease (CAD) has a higher prevalence and earlier onset in HIV-positive (HIV+) individuals [1]. In the era of combined antiretrovial therapy, serious non-AIDS complications (e.g., malignancies, liver disease, renal failure and major cardiovascular events) have been associated with a mortality risk twice that of AIDS-related events [2,3]. Specifically, data over a median follow-up period of 5.9 years from the HIV+ US Veteran Aging Cohort demonstrated a higher rate of incident acute myocardial infarction (AMI) among HIV+ compared to HIV- veterans (hazard ratio [HR] 1.48, 95% CI 1.27-1.72) [4]. Among patients followed by the Swiss HIV Cohort Study (SCHS), 6% of deaths were related to coronary artery disease (CAD) [5]. CAD progression has been linked to both internal and external causes of inflammation and endothelial stress [6,7]. Other studies have reported that HIV infection has worse outcomes in patients with CAD. In addition to the increased prevalence and earlier onset of incident AMI observed in HIV+ patients, this infection may also be associated with worse outcomes in those known to have CAD, such as recurrence of myocardial infarction or the need for coronary revascularization procedures [8-12].

These observations remain controversial, however, as they derive from small studies with heterogeneous inclusion criteria and substantial variation in the enrolled patients’ CAD therapy. Indeed, a recent prospective study revealed no differences in clinical outcome in incident AMI after one year of follow up between HIV+ and HIV- patients [13]. To further address this issue we used data pooled from two major nationwide cohorts in a nested cohort study to compare rates of all-cause mortality, recurrent AMI and cardiovascular-related hospitalization after one year of follow-up between populations of HIV+ and HIV- individuals hospitalised in Switzerland for an initial AMI.

## Methods

In a nested cohort study we compared clinical outcomes among adults in the Acute Myocardial Infarction in Switzerland (AMIS) registry and the Swiss HIV Cohort Study (SHCS).

### The Swiss HIV Cohort Study (SHCS)

The SHCS (www.SHCS.ch) has been enrolling HIV+ individuals aged 16 years or older in Switzerland since 1988; as of December 2011, a total of 17,349 persons were included [14]. All local ethics committees have approved the study; written informed consent is obtained from all participants. Participants receive care at outpatient clinics or physicians’ offices, where the following data are collected every six months: vital signs, a structured questionnaire with information on socio-demographic characteristics, illicit drug use, smoking status, co-morbidities and concomitant medications. In addition, a defined set of laboratory tests is performed including complete blood count, liver function tests, a lipid panel, hepatitis virus markers, CD4 cell counts and HIV viral load. Any laboratory results from intervening visits are also collected.

We included all SHCS participants who [1] were aged 18 years or older and [2] had HIV infection confirmed by enzyme-linked immunoabsorbent assay (ELISA) and Western blot who [3] experienced an incident AMI after registration in the SHCS between January 2005 and December 2011. Patients with a history of prior AMI were formally excluded. Diagnosis of myocardial infarction required either electrocardiographic evidence of ST-elevation (ST-elevation myocardial infarction (STEMI)) or non-ST-elevation myocardial infarction (NSTEMI) per ESC/ACC/AHA guidelines and in accordance with the Universal Definition of Myocardial Infarction [15-17]. Information on follow-up events (death, readmission) occurring within the first year after incident AMI was collected in a specific case report form sent to each affiliated SHCS centre.

### The Acute Myocardial Infarction in Switzerland (AMIS) registry

AMIS is a nationwide prospective registry with a 12-month follow-up of patients with AMI admitted to 81 hospitals (ranging from community institutions to large tertiary-care facilities) in Switzerland (www.amis-plus.ch) [18-20]. Data are collected via a standardized electronic or paper-based questionnaire and stored centrally at the Institute of Social and Preventive Medicine of the University of Zurich, then validated for plausibility and consistency. Use of the registry was approved by the Over-Regional Ethics Committee for clinical studies, the Swiss Board for Data Security and all cantonal ethics commissions [21].

From this parent cohort, we selected all HIV- men and women aged 18 years old and above who experienced an incident AMI after registration in AMIS between January 2005 and December 2011 [15-17]. Patients with a history of prior AMI were formally excluded. Diagnostic criteria used for AMI in this group were identical to those used in SCHS patients. The selected cut-off inclusion date was January 2005 coinciding with the generalised introduction of drug-eluting coronary artery stents into clinical practice [22,23]. AMI diagnoses were all validated after review of primary source data.

### Statistical analysis

#### Study endpoints

The primary endpoint was all-cause mortality; the secondary endpoint included a second AMI event or hospital readmission, all within one year of follow-up after the incident AMI. The diagnostic criteria used to define all cause mortality, AMI recurrence and hospital readmission was identical in both cohorts. Cardiovascular-related hospitalization was defined as a hospitalization event for reinfarction (STEMI/NSTEMI), unstable angina, need for coronary revascularization or stroke/TIA.

##### Descriptive analysis

Baseline demographic, clinical, laboratory and cardiac risk factor data were summarized according to HIV status using medians (interquartile range [IQR]) and the Wilcoxon rank-sum test for quantitative variables; percentages and chi-squared test for qualitative variables.

The use of platelet inhibitor recipients, antihypertensive drugs and lipid lowering drugs were extracted according to the following criteria: a) for patients registered in the SHCS: beginning these medications before the first AMI episode and until 6 month after the event; b) for patients registered in the AMIS registry: taking these as regular medication when the first AMI episode occurred or at hospital discharge.

##### Modelling

“Time to event” was defined as the time between the hospitalization date for the incident AMI and the date of death, cohort dropout or one year follow-up. Observation time was stopped at the time of the telephone interview when this was shorter that one year. The Kaplan-Meier method was used to calculate survival rate. Survival comparisons between groups were made using the log-rank test with an alpha threshold of 5 %. We developed three scenarios for deceased AMIS patients for whom information on exact date of death was lacking and for whom only the hospital discharge date and one-year follow-up call date were available: a) all deaths were assumed to have occured midway between the date of hospital discharge and the date of the one-year follow-up phone call (“average scenario”; noted HR_average_ in result section); b) all deaths were assumed to have occured on the date of hospital discharge (“worst scenario”; noted HR_worst_); and c) all deaths were assumed to have occured on the date of the one-year follow-up phone call (“best scenario”; noted HR_best_).

Hazard ratios were estimated to assess the association of HIV status with the occurrence of death using Cox proportional hazard models. For recurrent AMI and hospital readmission, logistic regression analysis with estimation of odds ratios (OR) on cumulative event information at one year was performed as specific dates were not reported in the AMIS registry. To identify potential confounding risk factors, analyses were first adjusted for gender, age and calendar year of AMI (M1). In addition, we performed 3 additional models adjusted for gender, age and calendar year of AMI to assess the specific contribution of smoking status (M2), hypertension (M3), and diabetes (M4), or the combination of smoking status, hypertension and diabetes (M5) on each of the 3 endpoints of interest. Due to an important number of missing values for the variables cholesterol (20%) and BMI (10%) and the small number of events, it was not feasible to employ more complex models, e.g., further adjustment for cardiovascular treatment, cholesterol, BMI or HIV-related variables. Statistical analyses were performed using Stata Release 12.0 (StataCorp, College Station, Texas, USA).

## Results

### Baseline characteristics

**Figure 1:**
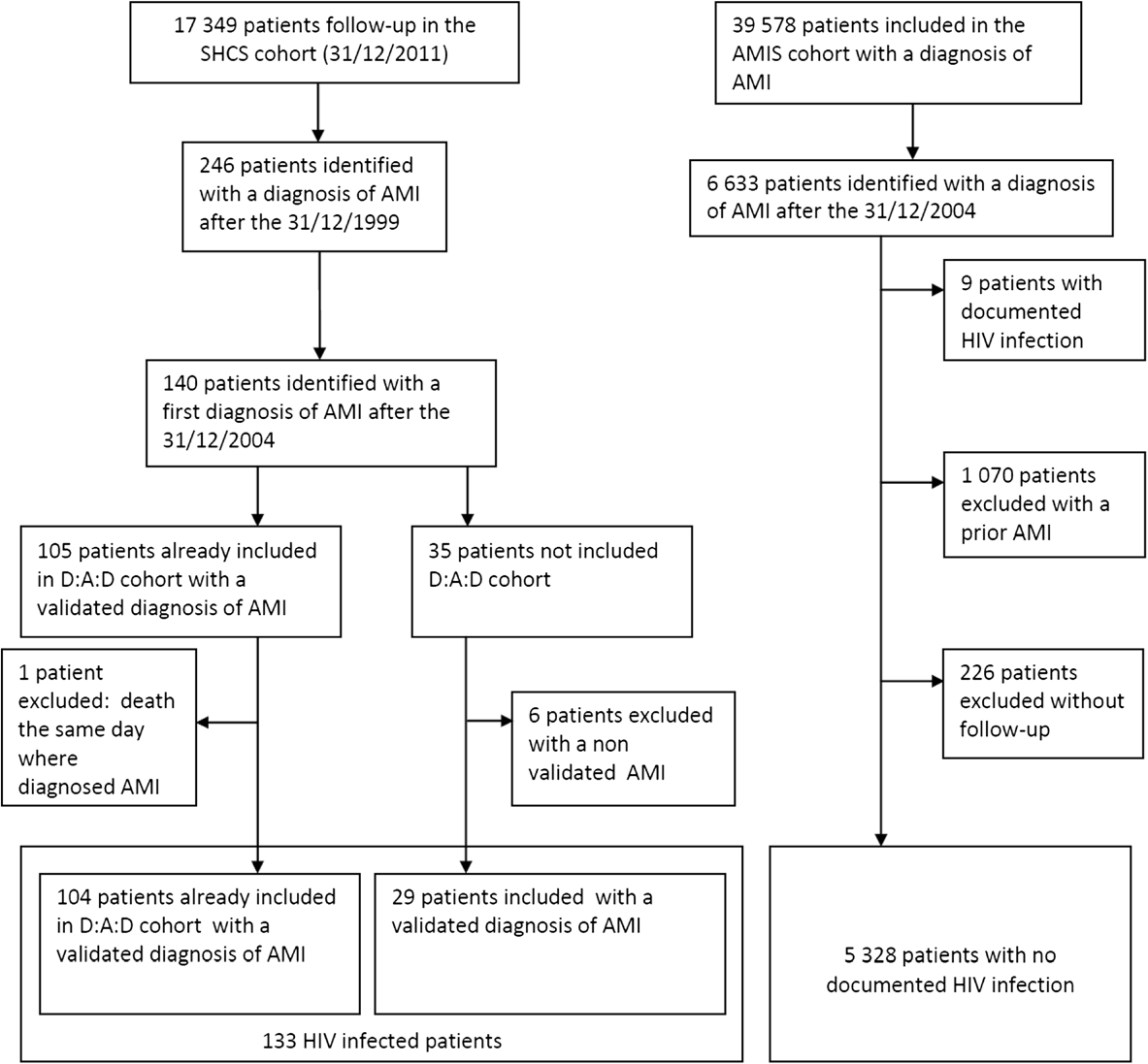
**Study flow chart.**

A total of 133 HIV+ and 5,328 HIV- individuals with incident AMI were enrolled (Figure 1). Compared with HIV – individuals, those who were HIV + were more often male (85.0% versus 72.2%, respectively (p = 0.001) and younger (median age 51 [IQR 46–57] years versus 64 years [IQR 55–74] (p < 0.0001) (Table 1).

**Table 1:** **Baseline characteristics of 133 HIV-positive subjects from the SHCS and 5328 HIV-negative subjects from the AMIS registry at the time of their first myocardial infarction**

	**SHCS**		**AMIS registry**		
	**HIV-positive subjects**		**HIV-negative subjects**		
	**Total**		**Total**		**P_values** ^**a**^
**Age, year**	133	51 (46–57)	5328	64 (55–74)	**<0.0001**
**Men**	133	113 (85.0)	5328	3845 (72.2)	**0.001**
**Weight (kg)**	121	71 (63–71)	5167	78 (69–88)	**<0.0001**
**Height (cm)**	133	174 (169–178)	4803	171 (165–176)	**0.0003**
**Body Mass Index (kg/m** ^**2**^ **)**	121	23. (21.6–26.1)	4801	26.5 (24.2–29.4)	**<0.0001**
**Blood pressure**					
Systolic (mmHg)	119	132 (120–149)	5273	140 (120–160)	**0.0034**
Diastolic (mmHg)	119	75 (83–90)	5270	81 (71–94)	0.9015
**Other cardiovascular risk factors**					
**Smoking history**	132		5094		**<0.0001**
- Active smokers		77 (58.3)		2009 (39.4)	
- Former smokers		23 (17.5)		1311 (25.7)	
- Non smokers		32 (24.2)		1774 (34.8)	
**Family history of CAD** ^**b**^	133	18 (13.5)	4717	1571 (33.3)	**<0.0001**
**Hypertension**	133	32 (24.1)	5323	3231 (60.7)	**<0.0001**
**Diabetes mellitus**	133	18 (13.5)	4734	739 (15.6)	0.569
**Biological parameters**					
Glycemia (mmol/l)	116	5.3 (4.7–6.0)	4920	7.1 (6.1–8.8)	**<0.0001**
Total cholesterol (mmol/l)	120	5.4 (4.5–6.3)	4078	5.4 (4.6–6.2)	0.952
HDL cholesterol (mmol/l)	120	1.1 (0.9–1.3)	3720	1.2 (1.0–1.5)	**0.005**
Serum creatinine (umol/l)	121	80 (69–93)	5158	81 (69–94)	0.779
**Discharge AMI diagnosis**	110		5328		**0.025**
STEMI		53 (48.2)		3135 (58.8)	
NSTEMI		57 (51.8)		2193 (41.2)	
**Current cardiovascular treatments**					
Platelets inhibitor recipients	133	120 (90.2)	5326	5169 (97.0)	**<0.0001**
Antihypertensive drugs	133	107 (80.5)	5327	4735 (88.9)	**0.002**
Lipid lowering drugs	133	115 (86.5)	5325	5028 (94.4)	**<0.0001**

Data are no. (%) of subjects or median (interquartile range).

^a^Wilcoxon rank-sum test for quantitative variables and chi square test for qualitative variable ^b^CAD: coronary artery disease.

Currently in the SHCS, over 80% of patients are receiving at least three antiretroviral drugs and 56% are treated with either cardiovascular or anti-diabetic drugs [24]. One hundred and forty HIV+ individuals were identified; 105 were already included in the D:A:D cohort (Data Collection on Adverse Events of Anti-HIV Drugs, www.chip.dk/DAD) [25] and had received a diagnosis of AMI according to the criteria described above; one of them was excluded because he died on the day of his first AMI. Thirty five patients were not included in the D:A:D cohort; 29 of these had their diagnosis confirmed by an experienced cardiologist based on available medical records. A total of 133 patients from the SHCS parent cohort were thus eligible for inclusion (Figure 1).

Between January 1, 2005, and December 31, 2011, a total of 6,633 patients with a diagnosis of AMI (STEMI, NSTEMI) were enrolled in the AMIS cohort. Of these, 5,328 were eligible for study inclusion, whilst 1,305 patients were excluded: by cross-checking each participant in both cohorts regarding demographic similarities such as age, sex, and geographic location we were able to identify 9 patients in the AMIS registry who corresponded to patients in the SHCS cohort and who were therefore HIV+. These patients were excluded to have two distinct patient cohorts, one including exclusively HIV+ and one only HIV- individuals. Furthermore 1,070 did not have a first episode of AMI, and 226 did not have complete follow-up (Figure 1).

Cardiovascular risk factors varied by HIV status. Compared with HIV - individuals, HIV + had a lower median BMI (23.8 kg/m^2^ [IQR 21.6-26.1] versus 26.5 kg/m^2^ [IQR 24.2-29.4] (p < 0.0001), lower systolic (but not diastolic) blood pressure (132 mmHg [IQR 120–149] versus 140 mmHg [IQR 120–160)] (p = 0.0034), higher likelihood of being active smokers (58.3% versus 39.4%, p < 0.0001), lower likelihood of being former smokers (17.5% versus 25.7%, p < 0.0001) and be more likely to be hypertensive or have a family history of CAD (24.1% versus 60.7%, p < 0.0001 and 13.5% versus 33.3%, p < 0.0001, respectively). Prevalence of diabetes mellitus was similar in the two groups. Among the HIV- AMIS population, 58.8% (3,135/5,328) received a discharge diagnosis of STEMI and the remaining 41.2% a diagnosis of NSTEMI, while among HIV+ individuals, 48.2% (53/110) were diagnosed with STEMI and the remaining 51.8% with NSTEMI. Serum glucose and high density lipoprotein (HDL) cholesterol were lower in HIV+ persons (5.3 mmol/l [IQR 4.7-6.0] versus 7.1 mmol/l [IQR 6.1-8.8], p < 0.0001) and 1.1 mmol/l [IQR 0.9-1.3] versus 1.2 mmol/l [IQR 1.0-1.5], p = 0.005, respectively). There were no significant differences between groups for total cholesterol and serum creatinine values. HIV - individuals were more likely to be treated with anti-platelet, antihypertensive or lipid lowering drugs after a first AMI than were HIV + patients (p < 0.0001 for anti-platelets and lipid-lowering drugs and p = 0.002 for anti-hypertensive drug).

Among the HIV+ group 49 patients (46%) were men who have sex with men (MSM) and 20 (15%) were intravenous drug users (IVDU). The median time from HIV diagnosis was 12 years (IQR 8–18) and a similarly long history of antiretroviral treatment (median 11.5 years); 120 (90%) were on antiretroviral therapy (ART) at study entry, with lengthy exposure to both nucleoside reverse transcriptase inhibitors (NRTIs; median10.9 years) and protease inhibitors (PIs; median 5.6 years). Ninety-five (77%) had viral loads below the detection level of 40 copies/ml; median CD4 count was 462 cells/mm^3^ (IQR 347–701; see Table 2).

**Table 2:** **HIV parameters at first AMI episode**

	**SHCS**	
	**HIV-positive subjects**	
	**N Total**	
Transmission risk category	133	
Heterosexual intercourse		42 (32)
Injection drug use		20 (15)
Men-who-have-sex-with-men		61 (46)
Other/unknown		10 (7)
Time since HIV diagnosis (years)	124	12 (8–18)
Patients with viral load <40 copies per mL	123	95 (77)
CD4 cell count per mm3	129	462 (347–701)
Antiretroviral treatment at the first episode of AMI		
Antiretroviral naïve	133	1 (1)
Not naïve, but not currently on antiretroviral drugs	133	12 (7)
On antiretroviral drugs	133	120 (90)
Antiretroviral regimen containing abacavir	120	51 (43)
Exposure to antiretroviral treatments (previous and current)		
Patients on PI (previous or current)	132	113 (85.6)
*Median duration of PI therapy (years)*	113	5.6 (3.5–9.4)
Patients under NNRTI (previous or current)	132	88 (86)
*Median duration of NNRTI therapy (years)*	88	3.5 (0.6–5.9)
Patients on NRTI (previous or current)	132	126 (95)
*Median duration of NRTI therapy (years)*	126	(6.7–15.1)

Data are no. (%) of subjects or median (interquartile range). PI, protease inhibitors; NNRTI, non-nucleoside reverse-transcriptase inhibitors; NRTI, nucleoside reverse-transcriptase inhibitors.

### One-year survival

Median follow-up duration for HIV- subjects was 392 days (IQR 375–414); for 4,953 (93%) of the patients we obtained follow-up by telephone call at 12 ± 3 months; for 92 (2%) it was before 9 months and for 283 (5%) it was beyond 15 months. In the HIV+ group, 128 (96%) patients had a follow-up of at least 12 months, while 5 were lost to follow-up. The exact date of death was known for all but 51 of the deceased HIV- individuals; data regarding repeat AMI and hospital readmission was missing for 394/5,328 (7.4%) and 349/5,328 (6.6%) patients, respectively.

Deaths occurred during the first year of follow-up in five (3.6%) of the HIV+ and 135 (2.5%) of the HIV- individuals; the cause of death was cardiac-related in three of the five HIV+ individuals, and in 44 of 61 HIV- individuals (Table 3). Kaplan Meier estimations of death probability at one year according to HIV status were not significantly different (3.8% vs 2.6%, p = 0.374, data not shown). However, at one year, HIV status adjusted for age, sex, calendar year of AMI, smoking status, hypertension and diabetes predicted patients’ survival (Table 3): HIV+ individuals with a first episode of AMI had a higher risk of death than their HIV- counterparts (HR 4.42, 95% CI 1.73-11.27). For the 51 deceased HIV- individuals with missing information on the exact date of the event, we conducted a sensitivity analysis to assess whether assigning the unknown date of death to the day of the one-year follow-up phone call or to the day of hospital discharge or would change the estimated risk and found the results were similar (HR_best_ 5.14, 95% CI 1.99-13.27 versus HR_worse_ 4.43, 95% CI 1.74-11.30, respectively).

**Table 3:** **Death, AMI recurrence, and re-hospitalization for cardiovascular reasons at 1-year follow-up**

	**SHCS**		**AMIS registry**		**M0**	**M1**	**M2**	**M3**	**M4**	**M5**
	**HIV-positive subjects**		**HIV-negative subjects**							
	**N Total**	**N (%)**	**N Total**	**N (%)**	**Crude HR or OR**	**Age, gender, calendar year Adjusted HR or OR**	**Age, gender, calendar year, smoking status Adjusted HR or OR**	**Age, gender, calendar year, hypertension Adjusted HR or OR**	**Age, gender, calendar year, diabetes Adjusted HR or OR**	**Age, gender, calendar year, smoking status, hypertension, diabetes Adjusted HR or OR**
					**(95% CI)**	**(95% CI)**	**(95% CI)**	**(95% CI)**	**(95% CI)**	**(95% CI)**
**Deaths:**										
- Cardiovascular death		3 (60.0)		44 (32.6)						
- Non-cardiovascular death		2 (40.0)		17 (12.6)						
- Unknown		0 (0)		74 (54.8)						
**Total death**	133	5 (3.6)	5328	135 (2.5)	1.50 (0.61; 3.65)	4.56 (1.80; 11.57)*	4.53 (1.78; 11.51)*	4.86 (1.91; 12.34)*	4.27 (1.69; 10.82)*	4.42 (1.73; 11.27)*
**Recurrent AMI**	133	4 (3.0)	4934	146 (3.0)	1.02 (0.37; 2.79)	1.20 (0.43; 3.34)	1.22 (0.44; 3.41)	1.23 (0.44; 3.44)	1.15 (0.41; 3.21)	1.16 (0.41; 3.27)
Re-hospitalization for cardiovascular reasons during the first year following the AMI	133	20 (15.0)	4979	1078 (21.6)	0.64 (0.40; 1.04)	0.66 (0.41; 1.08)	0.67 (0.41; 1.08)	0.69 (0.43; 1.13)	0.65 (0.40; 1.06)	0.68 (0.42; 1.11)
**Total**	133	20 (15.0)	5328	1199 (22.5)						

**M1:** Cox proportional hazard regression or logistic regression adjusted for gender, age and calendar year of AMI; **M2:** Cox proportional hazard regression or logistic regression adjusted for gender, age, calendar year of AMI. and smoking status; **M3:** Cox proportional hazard regression or logistic regression adjusted for gender, age, calendar year of AMI and hypertension; **M4:** Cox proportional hazard regression or logistic regression adjusted for gender, age, calendar year of AMI and diabetes; **M5:** Cox proportional hazard regression or logistic regression adjusted for gender, age, calendar year of AMI, smoking status, hypertension and diabetes; *p < 0.05.

HR, hazard ratio; OR, odds ratio.

HIV status remained a significant determinant of the occurrence of death at one year in distinct models integrating smoking status (M3), hypertension (M4), or diabetes (M5) with HRs in similar range in the 3 models (HR 4.53, 95% CI 4.58-11.51, HR 4.86, 95% CI 1.91-12.34, and HR 4.27, 95% CI 1.69-10.82 respectively).

### One-year clinical outcome

Recurrent AMI was noted in four (3.0%) HIV+ and 146 (3.0%) HIV- individuals. When adjusted for age, sex, calendar year of AMI, smoking status, hypertension and diabetes, there was no statistically significant difference between groups (M5, Table 3: OR 1.16; 95% CI 0.41-3.27). Hospitalization for cardiovascular-related events occurred in 20 (15%) HIV+ and 1,078 (21.6%) HIV- individuals. However, when adjusted for age, sex, calendar year of AMI, smoking status, hypertension and diabetes, this difference was not statistically significant (M5, Table 3, OR 0.68; 95% CI 0.42; 1.11).

## Discussion

In this retrospective nested cohort study we observed that deaths from all causes at one year after incident AMI were nearly 5 times more frequent among HIV+ than HIV- individuals. There was however no statistically significant difference in the risk of recurrent AMI or hospital readmissions in this group. HIV remains a significant risk factor predicting survival after a first incident AMI in this population, after adjustment for several classical risks factors.

Several other studies have examined cardiovascular outcomes in HIV-infected persons. Matezky et al. found that 24 HIV+ individuals who were age- and sex-matched with HIV- controls had a higher rate of reinfarction, recurrent cardiovascular events and revascularization independently of the type of their ART [9]. Another case–control study by Hsue et al. reported a higher rate of coronary restenosis after percutaneous coronary intervention in HIV+ compared to HIV- persons [10]. Boccara et al. reported long-term follow-up (median 41 months) from a case–control study comparing 27 HIV+ persons to 54 HIV- patients undergoing coronary artery bypass. Short-term outcomes (post-operative death, AMI, stroke, mediastinitis and reintervention) at 30 days were similar; however, the rate of occurrence of a first major cardiac event (MACE) was higher in the HIV+ group. This difference was largely attributable to the need for repeat PCI revascularisation of the native coronary arteries [11]. The same group confirmed these results in a more recent publication assessing one-year prognosis of 103 HIV+ and 195 HIV- patients after the occurrence of an acute coronary syndrome (ACS): despite similar angiographic features of coronary artery disease in both groups, recurrent ACS was 6 times more frequent in the HIV+ group [13]. Lorgis et al. Reported that after acute myocardial infarction, HIV status influences long-term risk, although th eshort-term risk is comparable to uninfected patients [26]. A recent meta-analysis of 11 studies including a total of 2,442 patients concluded that HIV+ patients admitted for ACS face a substantial short-term risk of death and a significant long-term risk of coronary revasculization and myocardial infarction, especially if receiving protease inhibitors [27].

Combining these results with our own findings suggests a higher risk of adverse outcomes after a first acute coronary event in HIV+ patients. During a certain era, this may have been in part due to lower revascularization procedure rates for HIV+ patients receiving AMI care, although there is has been an observed trend towards better access [12]. Indeed, an analysis by D:A:D investigators demonstrated an increased use of invasive cardiologic procedures post-AMI when comparing time periods between 1999 and 2002 and between 2009 and 2011. Thus the decreased mortality rate between these two periods (26.4% down to 8.2 %) may be in part due to the use of more efficient medical interventions, rather than in changes in patient characteristics [4,28]. The same trends have been observed in the AMIS Plus population between 1997 and 2011 [29]. Parcea et al. reported a 50% higher relative risk in hospital mortality from an AMI event in HIV+ individuals and observed that common related procedures (including left cardiac catheterization, coronary arteriography, and angiography of the left heart) occurred at significantly lower rates among HIV+ subjects [10].

Of note, the overall mortality rate reported by D:A:D investigators is higher than we found in our study [28]: in D:A:D, the short-term (one-month) mortality was as high as 20%, and over 33 months of follow-up a repeat AMI occured in 10% of the patients. A secular trend time period bias may partly explained this difference, as we only included patients with enrollment after 2005.

Indeed, HIV+ patients have a high-risk cardiovascular profile, largely due to the high prevalence of smokers, there being 60% among the SCHS particpants. Although our analyses were partially adjusted for confounding, the baseline characteristics of participants in the two cohorts differed significantly with respect to weight, BMI and systolic blood pressure: all of which were elevated in the AMIS HIV- group. A recent study showed that HIV infection is associated with a 50% increased risk of AMI beyond that explained by recognized Framingham cardiovascular risk factors, even in patients with sustained viral load suppression [30]. Cruz et al. reported that HIV+ AMI patients were younger and more often lacking in traditional cardiovascular risk factors than their HIV- counterparts [31]. In our cohort, patients from the SHCS were significantly younger and had lower rates of hypertension and diabetes mellitus (p < 0.0001). Even patients with sustained viral load suppression are at increased risk of incident AMI, raising the question of whether they may benefit from additional HIV specific prevention strategies such as earlier ART treatment. In the meantime, clinicians should continue to emphasize risk-factor modification strategies that have been proven effective, such as smoking cessation.

Residual HIV replication may possibly explain the increased cardiovascular risk and poorer outcome in HIV+ individuals. Findings from a recent nested case–control study suggest that CD4 T-cell nadir and a high current CD8 T-cell count are associated with an increased risk of AMI in HIV+ individuals [5]. Measures of subclinical coronary atherosclerosis using CT angiography showed a greater prevalence and greater extent of non-calcified plaque and stable calcified plaques in HIV+ men compared to HIV- men [32]. Interestingly, immune activation sCD163, a monocyte activation marker, is associated with subclinical disease (identified as low attenuation plaques) after adjustment for traditional CVD risks factors [33]. This might open new paths for interventions to reduce the risk of AMI in HIV+ patients.

Given that the number of cardiovascular procedures increased overtime, the number of patients in the D:A:D analysis receiving lipid-lowering drugs (44%), antiplatelet therapy (52%) or angiotensin-converting enzyme inhibitors (27%) was low, notwithstanding that these are standard of care in post-AMI management [28]. Our study confirmed that the prescription of standard post MI treatments at the time of AMI was surprisingly low, although better that the numbers reported in the D:A:D cohort, when compared to patients with no documented HIV infection in Switzerland. Boccara et al. already hypothesized that cardiologists may manage HIV-infected patients less aggressively due to the unknown prognosis of HIV infection itself. They observed that HIV- individuals were more likely to have recurrent revascularization guided by silent ischemia, partly explained by the higher use of stress testing found in this population [12]. Optimal dosing of drugs other than those specific for post-AMI treatment is also problematic in the general population; Arnold et al. found that after an AMI, nearly 85% of patients were discharged on beta-blocker doses that were substantially below the established effective dose, while 66% were discharged on insufficient doses of statins and ACE inhibitors [34].

In addition, drug-drug interactions involving cytochromes P450 (the metabolic pathway of many antiretrovirals, mainly boosted protease inhibitors) with inhibitors of the ADP-P2Y12 platelet receptors may lead to deleterious adverse effects: P2Y12 inhibitors such as prasugrel are not adequately bio-activated and thus are not effective [35]. Adverse events related to agents such as clopidogrel have also been well documented [36]. In summary, hypothetical reasons for worse short-term outcomes in HIV+ patients include less frequently use required cathterization procedures [10], ongoing HIV replication and inflammation [5], drug-drug interactions [29], and asymptomatic coronary disease at younger ages [31] leading to a late diagnosis of CAD.

Our study has several limitations. We did not have an exact date of the events of interest for some AMIS patients, although our “three scenarios” sensitivity analyses covering the worst- through best-case survival scenarios were consistent. The coding of the variable for hospitalization and co-medications was different in the two databases, complicating precise comparisons between the SHCS and the AMIS registry. We observed a tendency for fewer hospitalizations in AMIS patients, but this may be due to a reporting bias and misclassification in the SCHS, which documents hospitalizations with a higher degree of precision. These limitations also made a composite endpoint analysis difficult. Furthermore, because of a limited number of confirmed cardiovascular related deaths and a limited number of patients with recurrent AMI, expanding the model to further adjust for cardiovascular treatments, BMI or HIV-related variables, was not feasible.

## Conclusions

This study comparing clinical outcomes in individuals registered in two prospective national registries demonstrates an increased risk of death one year after initial AMI in HIV+ patients. These findings warrant further, long-term assessment, as HIV-infected individuals are increasing in prevalence and could benefit from better-informed cardiac risk stratification as well as targeted secondary prevention measures.
